# Study on Stamping–Bulging Process of Thin-Walled Superalloy Diaphragm for S-Shaped Bellows

**DOI:** 10.3390/ma17122829

**Published:** 2024-06-10

**Authors:** Zhubin He, Qingsong Zhao, Kun Zhang, Jian Ning, Yi Xu, Xianggang Ruan

**Affiliations:** 1 State Key Laboratory of High-Performance Precision Manufacturing, School of Mechanical Engineering, Dalian University of Technology, Dalian 116024, China; 15610565575@163.com (Q.Z.); ningjiannj@126.com (J.N.); xuyi1997@mail.dlut.edu.cn (Y.X.); ruanxg530@163.com (X.R.); 2 AVIC Shenyang Aircraft Corporation, Shenyang 110850, China

**Keywords:** ultra-thin bellows diaphragm, superalloy, FE simulation, stamping–bulging forming

## Abstract

A combined stamping–bulging forming process was proposed to achieve high-precision forming of large-diameter, ultra-thin-walled, superalloy welded S-type corrugated diaphragms. The underlying principle is to enhance the diaphragm’s forming accuracy by increasing the plastic deformation region and reducing springback. Using the ABAQUS version 6.14 finite element analysis software, finite element models were constructed for the stamping, hydraulic bulging, and combined stamping–bulging forming processes of the welded S-type metal corrugated diaphragms. A comparative analysis was conducted on the forming processes of the welded S-type metal corrugated diaphragms under the three forming methods, focusing on equivalent stress, distribution of wall thickness, and forming accuracy. This analysis determined the optimal forming process and the corresponding process parameters for superalloy welded S-type metal corrugated diaphragms. The results show that under a constant drawing force, as the bulging pressure increases, the plastic deformation of the straight sections of the diaphragm becomes more pronounced, resulting in improved shape accuracy. The combined stamping–bulging forming process guarantees the highest degree of shape accuracy for the diaphragm. The optimal process parameters were identified as a 30 t force and a 5 MPa pressure, with a maximum shape error of 0.02 mm. Concerning a plate thickness of 0.3 mm, the maximum deviation rate was found to be 6.7%, which represents a 30% improvement over traditional stamping processes. The maximum wall thinning rate was found to be 3.3%, a 1% reduction compared to traditional stamping processes, confirming the process’s feasibility.

## 1. Introduction

Corrugated tubes, characterized by their distinctive wavy structure, are rotary thin-walled shell bodies that exhibit excellent flexibility and bending capabilities. They serve functions such as displacement compensation and vibration absorption and are widely used in aerospace, petroleum, and chemical engineering industries. These tubes serve as essential components in the thermal fitting process to reduce thermal stress [1,2,3]. Metal bellows can be classified into distinct categories based on wave patterns, such as U-shaped, C-shaped, S-shaped, and Ω-shaped [4,5,6]. Corrugated tubes of the S-type are classified into two categories: the formed S-type and the welded S-type. Formed S-type corrugated tubes feature a large pitch and an S-shaped cross-section with a relatively small displacement compensation capacity. These components are typically manufactured using a hydraulic forming process and used to construct large heat exchangers. In contrast, welded S-type corrugated tubes have a smaller pitch and a larger displacement compensation capacity. These tubes are typically manufactured through film-forming and welding, resulting in a corrugated S-shaped cross-section. They are predominantly used as displacement compensation components in aviation engines.

As the flight speeds of aircraft continue to increase, the demands placed on aircraft engines for higher performance also continue to rise. Consequently, the welded S-type corrugated diaphragms, which are extensively used in aviation engine components, must be thinner, larger in diameter, and more complex in shape to achieve more significant maximum compression. This necessitates the implementation of even more precise techniques during the fabrication of the corrugated diaphragms [7,8,9]. Traditional stamping techniques to fabricate diaphragms frequently generate a considerable degree of springback after applying a cold stamping process. This phenomenon is often attributed to the high yield strength of superalloys. The attainment of high forming accuracy necessitates the implementation of iterative die adjustments, a process that is both onerous and time-consuming. Furthermore, the use of hot stamping necessitates the use of sophisticated machinery and molds, which results in a reduction in production efficiency and the occurrence of severe surface oxidation of the formed diaphragms. It can lead to inaccuracies in the dimensional specifications and subsequent welding processes. Although hydraulic bulging offers greater precision than stamping forming, it requires stringent equipment and sealing standards, and the significant wall thinning can affect the performance and fatigue life of the corrugated tube components [10,11,12].

Recent research on corrugated tubes primarily includes a few studies. Takuo N and colleagues [13] analyzed inclined-edge-welded S-type corrugated tubes’ deformation and fatigue life. They studied a welded metal corrugated tube with an inner diameter of 185 mm, an outer diameter of 230 mm, a wall thickness of 0.475 mm, and an axial height of 32.9 mm. Atmospheric and vacuum pressures were applied internally and externally to simulate the service environment. Through finite element simulation and experimental validation, they concluded that the maximum stress during compression in an inclined-edge corrugated tube forms a gradient on the outer straight section, effectively extending the life of the tube. Yuan and others [14] provided a mathematical description of reinforced S-type corrugated tubes and analyzed their mechanical properties, studying the mechanical characteristics under internal pressure, axial, and bending loads. Wang and colleagues [15] conducted numerical simulations and experimental studies on the axial hydraulic forming process of traditional large-amplitude S-type corrugated tubes made of 5A03 aluminum alloy. They investigated the effects of initial and final forming internal pressures and axial feed on the forming quality of the corrugated tubes with a wall thickness of 1.5 mm, a corrugation height of 39.37 mm, a corrugation width of 34 mm, and a wave crest fillet radius of 9.5 mm. Under optimal process parameters, they successfully formed S-type corrugated tubes with minimal wall thinning and high shape accuracy. Jooybari M B and colleagues [16] used 316L stainless steel as a material and, through finite element analysis and experimental validation, studied the effects of internal pressure load paths on the hydraulic forming of U-shaped corrugated tubes with a wall thickness of 0.5 mm, an internal diameter of 26 mm, an external diameter of 42 mm, and a wave width of 3 mm.

Current research on corrugated tubes primarily focuses on the mechanical properties and fatigue life analysis of welded S-type metal corrugated tubes, as well as the control of forming quality for integral hydraulic forming of S-type and U-type corrugated tubes. However, research into the precise forming of welded S-type metal corrugated diaphragms is lacking. Achieving the required forming accuracy for large diameter, ultra-thin, short wavelength GH4169 superalloy welded S-type metal corrugated diaphragms, a prerequisite for subsequent welding processes and service requirements, remains a challenge. This paper introduces a new composite forming process that merges stamping and bulging to form the diaphragms accurately. This study presents numerical simulations and experimental research on the forming process of GH4169 superalloy welded corrugated diaphragms. It analyzes the shape accuracy of the diaphragms under three different forming methods: stamping, hydraulic bulging, and a combined stamping–bulging process. The research also examines the impact of the drawing force and internal pressure during the combined stamping–bulging process on the diaphragms’ forming quality, which leads to the determination of optimal process parameters. The optimal parameters derived from the simulations were employed to fabricate the welded metal corrugated diaphragms, resulting in diaphragms that met the requisite criteria for wall thinning and shape accuracy. This confirms the feasibility of the process and achieves precise forming of the welded metal corrugated diaphragms.

## 2. Characterization of Thin-Walled Diaphragms for Welded S-Shaped Bellows

**Figure 1:**
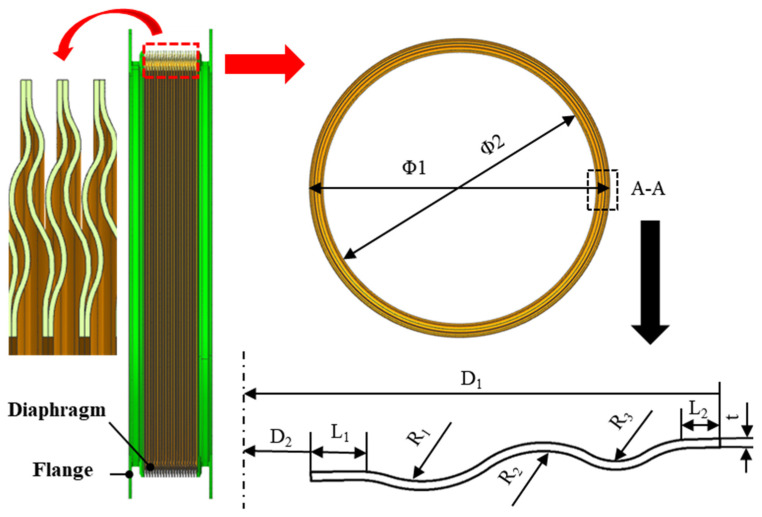
Welded S-type bellows and diaphragm.

The typical geometric model and cross-section of a welded, S-shaped, corrugated diaphragm are depicted in Figure 1. The diaphragm, which is annular in shape, has a cross-section forming an S-shaped corrugation, comprising three central arc segments and two straight segments at the ends. While the diaphragm possesses a large diameter, the forming region remains relatively narrow, and the cross-sectional shape experiences complex variations within a confined space. In addition, significant deformation occurs at the crests and troughs of the corrugations, and the forming accuracy of these small features directly affects the service performance of the corrugated tube. Previous studies have indicated that attaining uniform and sufficient deformation in small, intricate local features using conventional cold stamping presents a challenge, leading to subpar forming accuracy. On the contrary, hydraulic bulging necessitates substantial forming pressure and imposes stringent requirements on equipment and sealing.

## 3. Principle: Stamping–Bulging of Thin-Walled Diaphragm

**Figure 2:**
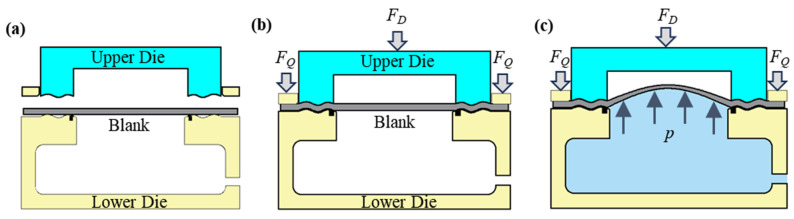
Schematic diagram of stamping–bulging forming principle of bellows diaphragm: (**a**) initial stage, (**b**) stamping stage, (**c**) bulging stage (*F_D_*-die closing force, *F_Q_*-blankholding force, *p*-bulging pressure).

To overcome the challenges of low forming accuracy and stringent equipment and sealing requirements in traditional thin-wall diaphragm forming, this study introduces a novel process that integrates stamping and bulging to form diaphragms. The core concept involves initially forming the diaphragm via stamping, followed by achieving comprehensive plastic deformation in the forming region through bulging. Figure 2 illustrates the principle of the combined stamping and bulging process for diaphragm formation. As depicted in Figure 2b, during the stamping phase, the blank holder is initially closed by a blank holder cylinder, which applies a specific blank holding force. Subsequently, the punch drives the upper die to close, applying a specific drawing force that induces plastic deformation in the blank, causing it to gradually conform to the die until it establishes full contact and forms a seal. As illustrated in Figure 2c, during the bulging phase, both the upper die and the blank holder remain stationary. Meanwhile, the liquid is injected into the lower die cavity, elevating the liquid pressure to a predetermined level. At this juncture, the fluid pressure primarily serves to adjust the stress state in the non-bulging region of the blank. During the unloading phase, the sealing ring disengages, the cavity pressure decreases, and both the blank holder and upper die retract, followed by the removal of the mold.

## 4. FE Analysis of the Forming Process of Welded S-Shaped Bellows Diaphragm

### 4.1. Material Properties

This study employed a 0.3 mm thick GH4169 superalloy material, a precipitation-hardened nickel-based alloy. Exhibiting high yield strength up to 650 °C and possessing excellent machinability and weldability, it is extensively utilized in aircraft engine components. To attain an axial compression ratio of 40% of the corrugated tube’s natural length and ensure extended service life, the diaphragm necessitates a complex shape with high dimensional precision. Consequently, high-precision forming of the diaphragm is imperative. This study conducted a simulation analysis of the forming process for a typical S-shaped corrugated tube diaphragm, as depicted in Table 1.

**Figure 3:**
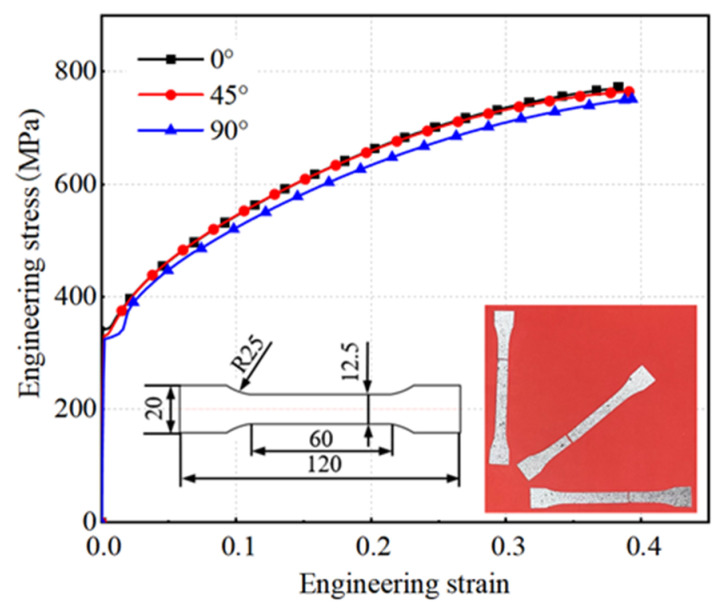
Engineering stress–strain curve of GH4169 superalloy.

Tensile tests were conducted along the rolling direction of the blank at angles of 0°, 45°, and 90°. As per GB/T 228.1-2021 [17], the test specimens were prepared with a rectangular cross-section and a proportionality factor of 5.56. Figure 3 presents the engineering stress–strain curves derived from the uniaxial tensile tests on the original GH4169 superalloy sheet, and Table 2 provides the corresponding mechanical properties of the blank.

### 4.2. FE Model of Different Forming Processes

#### 4.2.1. FE Model of Stamping Forming

**Figure 4:**
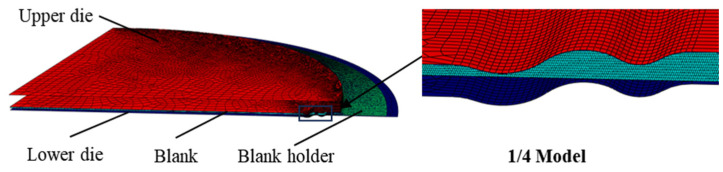
FE model of stamping forming.

The FE model employed for the stamping process comprised four elements: the blank, the lower die, the upper die, and the blank holder. The initial radius of the blank was set at 177 mm. Owing to the axisymmetric nature of the blank and the dies, an axisymmetric model was utilized for the numerical simulation. As depicted in Figure 4, the upper and lower dies, along with the blank holder, were discretized using shell elements, each with a mesh size of 0.5 mm. The blank material was represented as a deformable, isotropic, and homogeneous shell with a finer mesh size of 0.1 mm. The friction model employed was the Cullen friction model with a friction coefficient of 0.15. The material properties of the blank, as outlined in Table 2, adhered to the Hill 48 yield criterion for the material’s yield behavior.

The formation of the welded S-type corrugated diaphragm was simulated using an explicit dynamic analysis step, with a duration of 0.1 s. The interaction between the blank, the dies, and the blank holder was characterized as surface-to-surface contact. In this setup, the dies and the blank holder were discrete rigid bodies, while the blank was a deformable body. Therefore, the dies and the blank holder were set as the master surface, and the blank was set as the slave surface. The forming process commenced with the application of concentrated forces to the upper die, which governed its movement and facilitated die closure. The simulation was divided into two analysis steps. The initial step involved the application of an instantaneous concentrated force to the reference point of the blank holder, effectively reflecting its function, with the outer edge of the blank fully constrained. Simultaneously, a linear increase in the concentrated force was applied to the upper mold at 20 t, 30 t, and 40 t, respectively, and a finite element simulation was conducted for each set of concentrated forces. The subsequent step entailed the retraction of the upper die and the blank holder.

#### 4.2.2. FE Model of Hydraulic Bulging

**Figure 5:**
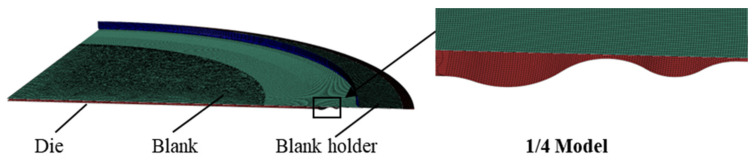
FE model of hydraulic bulging.

The FE model for hydraulic bulging consisted of three elements: the blank, the die, and the blank holder. The initial radius of the blank was set at 177 mm. As depicted in Figure 5, the attributes of the dies and blank, including the mesh division and contact type, correspond to those of the stamping forming finite element model. The hydraulic bulging process commenced with the application of uniform pressure to the upper surface of the blank. The forming process was bifurcated into two stages of analysis. The initial stage involved the application of an instantaneous concentrated force of 20 t to the reference point of the blank holder, thereby fully constraining the outer edge of the blank. Subsequently, a linear pressure was applied to the upper surface of the blank, escalating up to 85 MPa, until the blank completely conformed to the die. The subsequent stage entailed the retraction of the blank holder and the removal of pressure from the upper surface of the blank.

#### 4.2.3. FE Model of Stamping–Bulging Forming

**Figure 6:**
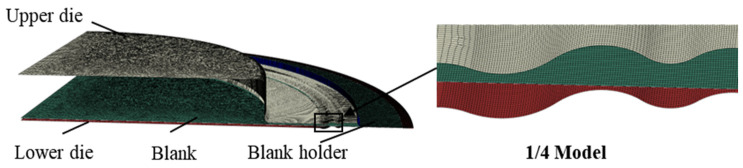
FE model of stamping–bulging forming.

The finite element model for the stamping–bulging forming process incorporated four components: the blank, the lower die, the upper die, and the blank holder. The initial radius of the blank was established at 200 mm, with an initial die gap of 2 mm. The upper die was characterized by a circular die cavity with a radius of 115 mm, a depth of 20 mm, and a fillet radius of 2 mm; the blank holder also possessed a fillet radius of 2 mm. As depicted in Figure 6, the attributes of the dies and blank, inclusive of the mesh division and contact type, were congruent with those utilized in the stamping forming finite element model. The stamping–bulging forming process was segmented into three analysis steps. The initial step involved the application of an instantaneous concentrated force of 20 t to the reference point of the blank holder, thereby fully constraining the outer edge of the blank. This was followed by the application of a linear concentrated force to the upper die until the dies achieved closure. The second step entailed the application of a linearly increasing uniform pressure within a circle of 117 mm radius on the lower surface of the blank to a predetermined value. The final step involved the retraction of the upper die and the blank holder.

During the stamping–bulging forming process, the quality of the diaphragm formation was primarily influenced by the drawing force and the internal pressure during bulging. Inappropriate parameter matching between these two factors can result in defects such as warping and cracking. To explore the optimal forming process parameters for the diaphragm in the stamping–bulging compound forming process, three drawing forces identical to those employed in the stamping process were selected. Additionally, three internal bulging pressures of 1 MPa, 3 MPa, and 5 MPa were chosen for investigation.

### 4.3. Deformation Behavior of Diaphragms under Different Forming Processes

#### 4.3.1. Stamping Forming

**Figure 7:**
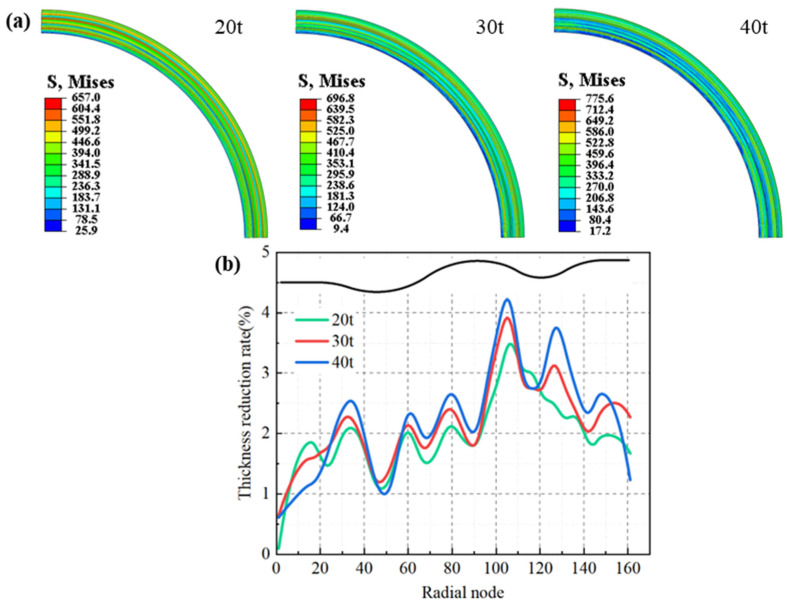
Distribution of equivalent stress and wall thickness reduction of stamping diaphragm: (**a**) equivalent stress, (**b**) wall thickness reduction.

In the stamping forming process, a concentrated linear force was exerted on the upper die to close the mold, thereby inducing deformation in the blank. Upon full closure of the mold, the distribution of equivalent stress and the thinning rate in the formed area of the blank are depicted in Figure 7.

The circumferential distribution of the equivalent stress across the blank was observed to be uniform, with the maximum equivalent stress consistently manifesting in the right arc segment and lesser equivalent stress in the left arc segment. As the tension escalated, the maximum equivalent stress correspondingly increased. When the drawing force attained 40 t, the maximum equivalent stress in the formed region of the blank peaked at 775.6 MPa. However, the area of high-stress distribution diminished, leading to a more uniform stress distribution. The maximum thinning of the blank transpired at the junction of the right arc and the crest, with the thinning rate gradually decreasing from the crest to the trough. The thinning in the straight sections was minimal, while the deformation in the arc segments resembled bending deformation. Studies have indicated that the smaller the relative bend radius R/t (where R represents the bend radius and t denotes the plate thickness), the smaller the ratio of the plate thickness post-bending to its original thickness, thereby resulting in greater thinning of the plate. Given that the radius of curvature of the right arc segment was significantly smaller than that of the left, the wall thinning was more pronounced on the right than on the left arc segment, with the maximum wall thinning occurring on the right arc segment. As the drawing force amplified, the maximum wall thinning rate also increased. For all three drawing forces, the maximum wall thinning rate satisfied the requirement of being less than 5%. When the drawing force was 40 t, the maximum wall thinning rate reached 4.2%. At a drawing force of 20 t, the maximum wall thinning rate was the lowest, but the distribution of wall thickness was not as uniform as it was when the drawing force was 30 t.

**Figure 8:**
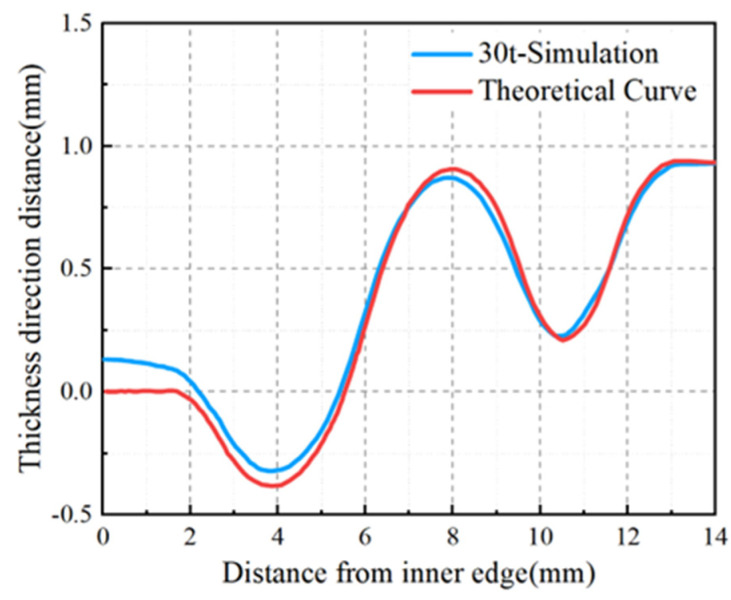
Stamping forming finite element simulation results forming accuracy.

The outcomes of the finite element simulation are represented as point cloud data. For a precise analysis of forming accuracy, it is imperative to extract and compare the coordinates of points along the diameter. Given the axisymmetric nature of the blank, any diameter can be chosen for comparison with the theoretical curve. Following an extensive analysis of the equivalent stress distribution and the maximum wall thinning rate, it was determined that a drawing force of 30 t would yield a more uniform stress distribution and wall thickness, thereby meeting the specified requirements. Any further increase in the drawing force would proportionally increase the maximum wall thinning rate. Consequently, a drawing force of 30 t was deemed optimal for the stamping process. The forming accuracy of the finite element simulation results under a 30 t drawing force is illustrated in Figure 8. The inner edge of the diaphragm was designated as the origin, with the radial distance from the inner edge defined as the X-value, and the distance from the inner edge in the thickness direction defined as the Y-value. The digital model derived from the simulation aligns with the theoretical model, particularly the right straight section. The results indicate that the finite element simulation closely mirrored the theoretical curve, with a slight springback in the arc segments and the greatest deviation in the straight sections, reaching a peak of 0.14 mm. Using a sheet thickness of 0.3 mm as a reference, the deviation rate was 46.7%. A larger deviation rate significantly escalates the complexity of subsequent welding. When the corrugated tube components are operational, substantial dimensional deviations can impact their performance and curtail the service life of the corrugated tubes.

#### 4.3.2. Hydraulic Bulging

**Figure 9:**
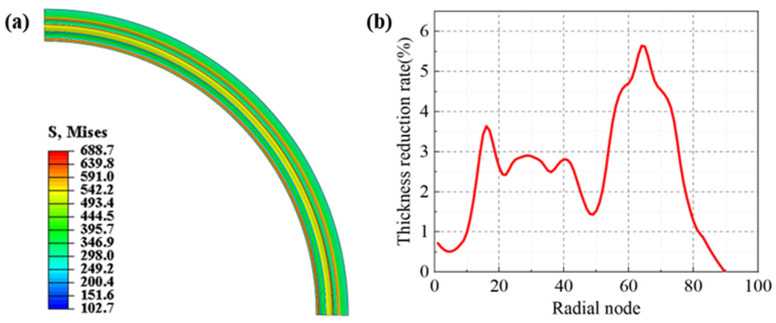
Distribution of equivalent stress and wall thickness reduction of hydraulic bulging diaphragm: (**a**) equivalent stress, (**b**) wall thickness reduction rate.

Hydraulic bulging was accomplished by applying uniform pressure to the top of the blank, culminating in full contact when the pressure escalated to 85 MPa. The distribution of equivalent stress and the rate of wall thinning in the formed region of the blank is illustrated in Figure 9. It was observed that the circumferential distribution of the equivalent stress across the blank was uniform, with the apex stress occurring at the junctions of the straight and curved sections, peaking at 688.7 MPa. The stress initially decreased and then ascended towards the crest from these junctions, with the nadir stress at the trough. The wall thickness distribution of the blank was uneven, with the maximum thinning transpiring at the junction of the crest and the right arc, reaching a peak thinning rate of 5.6%. The wall thickness along the left arc section was more uniform and thinner than that along the right arc. Thinning in the straight sections was minimal, almost negligible, and the thinning trend aligned with that of the stamping process. However, the maximum wall thinning rate surpassed 5%, which is the threshold for the corrugated tube components, as the maximum wall thinning rate of the diaphragm should not exceed 5%. If the wall thinning rate of the corrugated tube diaphragm surpasses the required value, the stress concentration in the area of maximum thinning can instigate cracks and holes during service, thereby reducing the lifespan of the component.

#### 4.3.3. Stamping–Bulging Forming

Prior studies suggest that the stamping process yields diaphragms with relatively low shape accuracy. While the hydraulic bulging process achieves superior shape accuracy, it necessitates substantial bulging pressure, which is challenging to attain with standard equipment, and requires exceptionally high sealing performance. Moreover, there is excessive thinning in the forming region, with the maximum wall thinning rate surpassing the specified requirements. This paper introduces a stamping–bulging compound forming process designed to enhance forming accuracy while also adhering to the maximum wall thinning rate specifications. The process is bifurcated into two steps: the initial step involves stamping to achieve pre-forming, and the subsequent step involves bulging in the non-forming central area of the blank post-stamping. The bulging step applies tensile forces to the edges of the bulging region, altering the stress state in the forming area of the blank. By transitioning part of the stress state from compressive to tensile stress, the process augments the distribution of tensile stress in the forming area, facilitating more comprehensive plastic deformation and thereby suppressing springback, resulting in improved forming accuracy.

**Figure 10:**
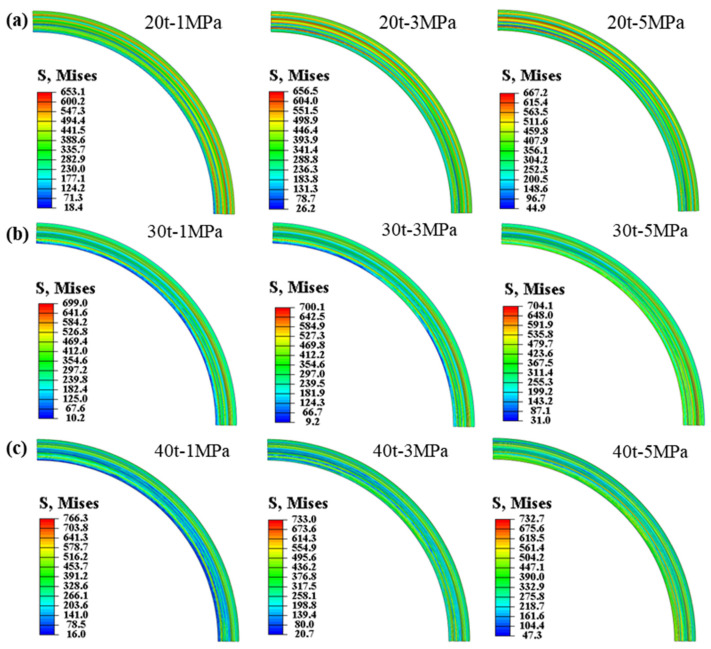
The equivalent stress distribution of the diaphragm in the stamping–bulging forming process: (**a**) drawing force 20 t, (**b**) drawing force 30 t, (**c**) drawing force 40 t.

The distribution of equivalent stress in the forming region during the stamping–bulging compound forming process at varying drawing forces and internal bulging pressures is depicted in Figure 10. It can be discerned that the internal bulging pressure exerts a minimal effect on the stress distribution in the forming region of the blank. At a draw force of 20 t, an escalation in internal bulging pressure results in a less uniform distribution of equivalent stress in the forming region of the diaphragm. Conversely, at 30 t and 40 t, an increase in the internal bulging pressure engenders a more uniform distribution of the equivalent stress in the forming region of the diaphragm. The impact of internal bulging pressure on the magnitude of the equivalent stress in the forming region is correlated to the magnitude of the drawing force. At 20 t and 30 t, the maximum equivalent stress in the forming region of the diaphragm marginally increases as the internal bulging pressure escalates. However, at a draw force of 40 t, the maximum equivalent stress diminishes rather than increases as the internal bulging pressure rises, declining from 766.3 MPa to 732.7 MPa.

**Figure 11:**
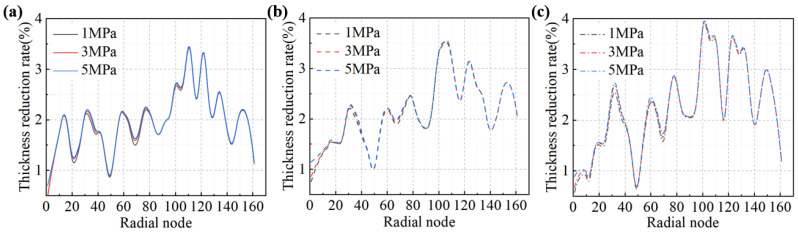
Thickness reduction distribution of diaphragm in the stamping–bulging forming process: (**a**) drawing force 20 t, (**b**) drawing force 30 t, (**c**) drawing force 40 t.

The distribution of wall thinning in the forming region during the stamping–bulging compound forming process at varying drawing forces and internal bulging pressures is depicted in Figure 11. The internal bulging pressure exerts a negligible impact on the wall thickness of the forming region, aligning with the wall thinning distribution observed during the stamping process under the corresponding drawing forces. During the bulging phase of the stamping–bulging compound-forming process, the non-forming central area of the blank undergoes bulging, thereby affecting the wall thickness of the non-forming region to modulate the stress state in the forming region. This regulation facilitates more comprehensive plastic deformation in the forming region, mitigating springback and enhancing the accuracy of the diaphragm shape.

The simulation outcomes revealed that at a drawing force of 20 t, an escalation in the internal bulging pressure resulted in less uniform stress distribution in the forming region. Conversely, at a drawing force of 30 t, an increase in internal bulging pressure engendered a more uniform stress distribution, accompanied by modest wall thinning and a relatively uniform wall thickness distribution. However, when a forming process of 40 t-5 MPa was employed, excessive wall thinning was observed. Based on these findings, three sets of parameters—30 t-1 MPa, 30 t-5 MPa, and 40 t-3 MPa—were provisionally selected for forming accuracy analysis by comparing the diaphragm shapes with the theoretical model curves.

**Figure 12:**
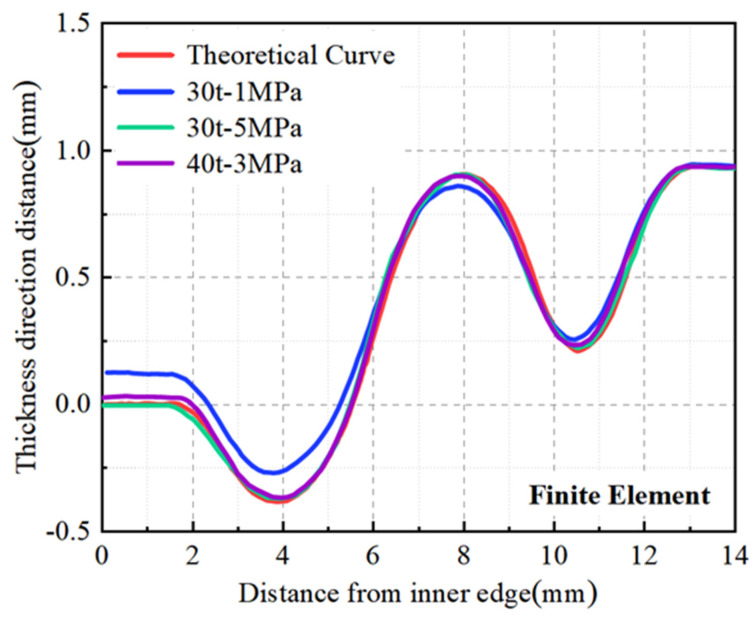
Diaphragms formed by different parameter simulations.

The point cloud coordinates derived from the simulation were extracted, and the right straight sections were aligned. The analysis of the shape accuracy of the diaphragms obtained with different parameters is depicted in Figure 12. It can be discerned that the diaphragm formed at 30 t-1 MPa deviates significantly from the theoretical model, with a maximum deviation of 0.12 mm on the left straight section, resulting in a maximum deviation rate of 40% based on a plate thickness of 0.3 mm. The 30 t-5 MPa and 40 t-3 MPa diaphragms are closer to the theoretical model, with the 30 t-5 MPa diaphragms being the closest, with a maximum deviation of 0.03 mm at the junction of the left straight section and the arc segment, corresponding to a maximum deviation rate of merely 10%.

The stamping–bulging forming process addresses the challenges associated with both stamping and hydraulic bulging forming. It yields diaphragms with superior dimensional accuracy than can be achieved by stamping alone while alleviating the stringent equipment and sealing requirements of hydraulic bulging. Furthermore, the maximum wall thinning rate was lower than that of diaphragms formed by hydraulic bulging, as illustrated in Table 3. Enhanced forming accuracy facilitates subsequent welding steps, enabling better alignment with the welding fixtures, and augmenting the welding precision and dimensional accuracy of the corrugated tube components. Amplifying the minimum wall thickness fosters a more uniform wall thickness distribution, thereby prolonging the service life of the corrugated tube components during operation.

## 5. Experimental Verification

Drawing upon the simulation results, a drawing force of 30 t was employed for the stamping experiment of the welded S-type corrugated diaphragm. Additionally, three sets of parameters—30 t-1 MPa, 30 t-5 MPa, and 40 t-3 MPa—were utilized for the corrugated tube diaphragm stamping–bulging experiment.

**Figure 13:**
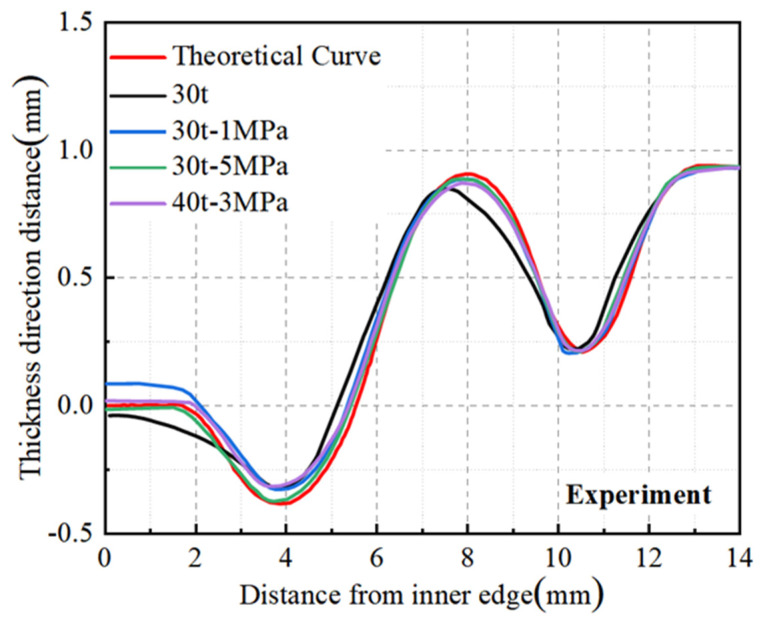
Diaphragms formed by different processes and parameters.

A GOM 3D scanner was utilized to capture the digital model of the diaphragm, which was subsequently processed in Origin software version 2021 to derive the shape curves of both the experimentally produced diaphragm and the theoretical model. This comparison aimed to evaluate the shape accuracy of diaphragms fabricated using different processes and parameters. As depicted in Figure 13, the results aligned with the simulation analysis, indicating that the diaphragm formed solely by stamping exhibited lower accuracy with a significant deviation from the theoretical curve. In contrast, the diaphragms formed by the stamping–bulging process demonstrated superior forming accuracy, with the 30 t-5 MPa parameter set yielding the closest alignment to the theoretical model and thus the highest forming accuracy.

**Figure 14:**
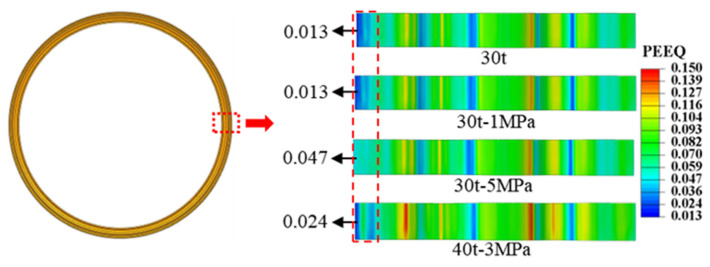
The radial equivalent plastic strain distribution of the diaphragm obtained by different processes and parameters.

As illustrated in Figure 13, the diaphragm formed solely by 30 t stamping exhibited a maximum deviation of 0.11 mm from the theoretical curve, with a maximum deviation rate of 36.7% based on a sheet thickness of 0.3 mm. Figure 14 reveals that the left straight section of the 30 t stamped diaphragm underwent inadequate plastic deformation, leading to significant springback and poor forming accuracy upon mold removal due to elevated residual stresses. In contrast, the diaphragm formed by the 30 t-5 MPa stamping–bulging process, as depicted in Figure 13, has a maximum deviation of 0.02 mm from the theoretical curve, with a maximum deviation rate of 6.7%, signifying a 30% enhancement in forming accuracy over conventional stamping. Figure 14 demonstrates that the 30 t-5 MPa stamping–bulging process imparts tensile stress to the left straight section of the forming area during the bulging phase following stamping. This results in more comprehensive plastic deformation in the left straight section, a substantial reduction in residual stress, and diminished springback, thereby significantly improving forming accuracy. Given these results, the stamping–bulging process is deemed the optimal forming technique for the typical welded S-type corrugated diaphragm, with the optimal process parameters identified as 30 t-5 MPa.

**Figure 15:**
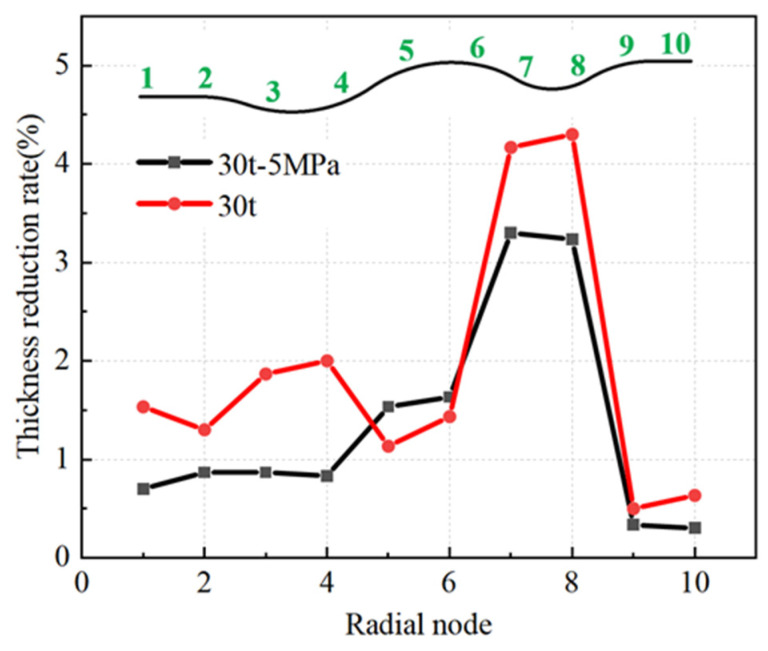
Thickness reduction rate distribution.

An assessment of the wall thinning status was conducted on the diaphragms formed using the optimal process parameters of 30 t-5 MPa for the stamping–bulging process and the 30 t stamping process alone, as portrayed in Figure 15. The most noticeable wall thinning occurred in the smaller radius arc segment on the right side of the diaphragm. The maximum wall thinning rate for the diaphragm formed by stamping alone was 4.3%, while that for the diaphragm formed by the stamping–bulging process was 3.3%, a decrease of 1% compared to the stamping process alone. Both maximum wall thinning rates were below 5%, satisfying the performance requirements for corrugated tube components.

## 6. Conclusions

In this paper, a stamping–bulging forming process was developed to improve the shape accuracy of ultra-thin S-shaped diaphragms. The main conclusions are as follows:

(1) This study addresses the critical challenge of achieving high forming accuracy for large-diameter, thin-walled S-shaped diaphragms made from GH4169 superalloy by introducing a novel stamping–bulging forming process. Finite element simulations conducted using ABAQUS revealed the deformation behavior during the stamping, hydraulic bulging, and combined stamping–bulging processes. Experimental validation confirmed the feasibility and effectiveness of this innovative technique.
(2) The stamping–bulging forming process emerged as the optimal method for diaphragm fabrication. This process modifies the stress state in the blank’s forming region, resulting in more complete plastic deformation at the diaphragm’s inner edge and significantly reducing springback. Consequently, forming accuracy was substantially improved. Optimal parameters were determined to be 30 t-5 MPa, achieving a maximum shape error of 0.03 mm and a deviation rate of only 10%, reflecting high precision. Additionally, the maximum wall thinning rate was limited to 3.6%, meeting the stringent accuracy requirements for corrugated tube components.
(3) Experiments confirmed the reliability of the simulation analysis and showed that the stamping–bulging forming process produces diaphragms with higher forming accuracy. The maximum shape error of the diaphragms was 0.02 mm, with a maximum deviation rate of only 6.7%, representing a 30% improvement in precision over traditional stamping. The maximum wall thinning rate was 3.3%, a reduction of 1% compared to stamping alone, and in both cases, the maximum wall thinning rate remained below 5%.

## Figures and Tables

**Table 1:** Typical diaphragm size parameters(mm) according to Figure 1.

Φ_1_	Φ_2_	L_1_	L_2_	R_1_	R_2_	R_3_	t
304	276	1.5	1	3.6	3.5	1.8	0.3

**Table 2:** Mechanical properties of GH4169 superalloy plates.

	0°	45°	90°
Yield Strength (MPa)	342.9	331.8	325.5
Tensile Strength (MPa)	772.8	765.1	751.5
Elongation (%)	0.383	0.395	0.393
Lankford Coefficient	0.932	1.156	1.136

**Table 3:** Comparison of forming effects of different processes.

Forming Method	Thickness Reduction	Shape Accuracy
Stamping forming	Less	Low
Hydraulic bulging	More	High
Stamping–bulging forming	Less	High

## Data Availability

Data are contained within the article.
